# The Effects of Dietary Inclusion of Bilberry and Walnut Leaves in Laying Hens’ Diets on the Antioxidant Properties of Eggs

**DOI:** 10.3390/ani10020191

**Published:** 2020-01-22

**Authors:** Arabela Elena Untea, Iulia Varzaru, Tatiana Dumitra Panaite, Teodor Gavris, Alexandra Lupu, Mariana Ropota

**Affiliations:** Chemistry and Nutrition Physiology Department, National Research and Development Institute for Biology and Animal Nutrition, Calea Bucuresti, No.1, 077015 Balotesti, Ilfov, Romania; iulia_maros@yahoo.com (I.V.); teogavris@gmail.com (T.G.); alexandra.fustei@yahoo.com (A.L.); mropota@yahoo.com (M.R.)

**Keywords:** bilberry leaves, walnut leaves, laying hens, egg yolk, antioxidant nutrients, oxidative stability

## Abstract

**Simple Summary:**

Eggs are considered common dietary sources of n-3 polyunsaturated fatty acids (PUFA), but they are susceptible to quality deterioration. In order to reduce oxidation, the best strategy is the addition of antioxidants to hens’ diets. Walnut and bilberry co-products, rich in phenolic compounds, are produced in agricultural industries, and are a valuable source of antioxidants for animal nutrition. The dietary inclusion of bilberry and walnut leaves as natural sources of antioxidants in poultry diets led to the accumulation of nutrients with antioxidant activity in the egg yolk, and the susceptibility of the yolk to lipid peroxidation during storage improved. The results of the present study showed that including bilberry and walnut leaves in laying hens’ diets enhanced the oxidative stability of the egg yolk and retarded the lipid peroxidation process during storage.

**Abstract:**

The purpose of this study was to examine the effects of dietary inclusion of walnut and bilberry leaves (WL and BL), as sources of natural antioxidants in poultry diets, on the enrichment of antioxidant nutrients in the egg yolk and on the susceptibility of the yolk to lipid peroxidation during storage. The experiment was conducted on 32-week-old TETRA SL LL laying hens, which were assigned to three dietary treatments with 30 birds each. Each treatment was replicated 10 times with three chicks per replicate pen. Experimental dietary treatments differed from control diet (C) by addition of 0.5% BL supplement or 1% WL supplement. The phytoadditives were not significant contributors to the productive parameters. The addition of BL and WL in laying hens’ diets significantly increased the total polyphenol content, and the zinc, lutein and zeaxanthin concentrations in the egg yolks. In regards to the oxidative stability parameters, a significant decrease in the concentrations of primary oxidation products formed in the egg yolk of experimental groups was seen, proving an efficient inhibition effect of the phytoadditives on peroxyl radical formation. A significant correlation was observed between primary oxidation products and total polyphenol content of the egg yolks, where zinc, lutein and zeaxanthin are the bioactive compounds that inhibit the formation of secondary oxidation products.

## 1. Introduction

Consumer acceptance defines the quality of food in terms of taste, aroma and appearance characteristics. Consumers have been expressing their concern about the safety of additives since the 1990s. In addition, the demand for sustainable sources and environmentally friendly production has led to an intensive search for food additives that are organic/natural, with familiar names [1]. Eggs constitute an important source of macronutrients and micronutrients, such as proteins, lipids, vitamins and minerals [2]. They are considered a common dietary source of n-3 polyunsaturated fatty acids (PUFA), but they are susceptible to quality deterioration [3,4].

In order to reduce oxidation, the best strategy is the addition of antioxidants in hens’ diets. Commonly, synthetic antioxidants (BHA, BHT, EDTA or vitamins (ascorbic acid, alpha tocopherol)) are used to inhibit oxidation, but many herbs and spices also contain antioxidant components [1]. The main antioxidants from plants are phenolic compounds, carotenoids and vitamins. Antioxidants counteract oxidation by protecting lipids from oxidation initiators or by stalling the propagation phase [5].

Preventive antioxidants act via different pathways including pro-oxidant enzyme inhibition, singlet oxygen deactivation, chelation of transition metals, etc. [5]. As examples: carotenoids are the most efficient molecules for singlet oxygen quenching; zinc does not directly attack free radicals but is important in the prevention of their formation due to its antagonistic effect on the catalytic properties of redox active transition metals (Fe, Cu) [6]; and vitamin E and polyphenols prevent the formation of lipid hydroperoxides by breaking the chain of lipid peroxidation in cell membranes [7].

Walnut (*Juglans regia L.*) is considered a priority plant by the food and agriculture organization (FAO) and is a strategic species for human nutrition [8]. Walnut co/by-products, rich in phenolic compounds, are produced in agricultural industries and are a valuable source of antioxidants for animal nutrition. Walnut leaves are considered to be a rich source of phenolic compounds, and are responsible for some of their therapeutic properties like their anti-allergic, anti-inflammatory, antiviral, antiproliferative and anticarcinogenic properties [9].

Bilberry (*Vaccinium myrtillus L*.) is a perennial wild shrub, belonging to Vaccinium spp., and is a good source of natural phenolic compounds, such as anthocyanins, which provide the natural pigmentation of fruits and exhibit cardio and neuroprotective effects, and anti-inflammatory and anticarcinogenic properties [10].

The purpose of this study was to examine the effects of the dietary inclusion of walnut and bilberry leaves (sources of natural antioxidants) in poultry diets on the accumulation of nutrients with antioxidant activity in the egg yolk, and on the susceptibility of the yolk to lipid peroxidation during storage.

## 2. Materials and Methods 

The experiment complied with Directive 2010/63/EU on the protection of animals used for scientific purposes and the experimental procedures were approved by the Ethical Commission of National Research and Development Institute for Biology and Animal Nutrition.

### 2.1. Experimental Design 

A four-week experiment was conducted on 32-week-old TETRA SL laying hens, which were assigned to three dietary treatments with 30 birds each. Dietary treatments, administered throughout the entire experimental period, were: (1) Control diet (C) containing a corn–soybean diet with no added phytoadditive supplement; (2) BL diet containing the previous C diet with an additional 0.5% bilberry leaves supplement; and (3) WL diet containing the previous C diet with an additional 1% walnut leaves supplement (Table 1).

The animals were housed in digestibility pens (Zucammi batteries (Z.M.E.C 50-model 2012) dimensioned according to the sanitary-veterinary norms regarding the minimum standards for the protection of laying hens (height of front = 455 mm, height of back = 375 mm, total depth = 550 mm, height between tiers = 582 mm, and tilt = 14%) in a poultry experimental hall with controlled environmental conditions (average temperature/total period 23.08 ± 0.98 °C; humidity 66.35 ± 5.68%; ventilation/hen 1.70 ± 0.14%). The light regimen was adequate for the age of the hens (16 h light/8 h darkness). Each treatment was replicated 10 times with three chicks per replicate pen. The birds were fed *ad libitum* and had free access to water. No medical care program was applied during the experimental period (the vaccination program was performed by the producer according to the TETRA SL LL Commercial Management Guide).

At the end of the experiment, all eggs laid by hens were considered and 18 eggs from each group (representing all treatment replicates) were randomly collected and analyzed for oxidative stability-influencing parameters (antioxidant capacity, total polyphenols, vitamin E, vitamin A, xanthophylls (lutein, zeaxanthin), zinc) and fatty acid composition. For the shelf life determinations, egg samples (18 eggs/group) were collected randomly at the end of experiment and stored at 16 ± 0.7 °C, for 28 days. After 28 days of storage, the egg samples were analysed for primary and secondary oxidation products. Throughout the experimental period, the feed intake, feed conversion ratio, egg production, egg weight and laying percentage were recorded on a daily basis. The laying percentage was calculated as an average daily ratio between the number of laid eggs and the number of hens. The feed conversion ratio was calculated as an average daily feed intake and the weight of daily laid eggs. The egg weight used for evaluation of productive parameters was the weight of eggs from the daily collection.

### 2.2. Plant Materials and Tissue Extracts

The plants used in the experiment were obtained from local pharmacies, and then dried, grounded and packed. The choice of the plant mixture, which was bilberry (*Vaccinium myrtillus L.*) leaves and walnut (*Juglans regia L.*) leaves, was based on their known properties and use in traditional medicine.

The plant extracts used for the estimation of total phenolic content and total antioxidant capacity (TAC) was obtained by extracting 1 g of dried plant powder in 10 mL of methanol, which was kept on a rotary shaker for 24 h in the dark. The extract was centrifuged at 1500 g for 10 min, and the supernatant was considered for analysis.

The yolk extracts used for the estimation of total phenolic content and total antioxidant capacity (TAC) were prepared as described by [11] with slight modifications. Briefly, five grams of fresh yolk was homogenized, 40 mL of 0.05 M phosphate buffer (pH 7) was added, and the mix was centrifuged at 12,000 g for 60 min. The supernatant obtained was used for analytical determinations.

### 2.3. Chemical Analysis 

#### 2.3.1. Zinc Determinations

Zinc concentrations were determined in the plants, diets and egg yolk samples using flame atomic absorption spectrometry (FAAS), as described by [12], after microwave digestion, by using Thermo Electron—SOLAAR M6 Dual Zeeman Comfort (Cambridge, UK) equipment.

#### 2.3.2. Content of Total Polyphenols (TP)

The total phenol content of all extracts was measured spectrophotometrically according to the Folin–Ciocalteu method, as described by [13], with slight modifications. Briefly, the extract samples (0.5 mL of different dilutions) were mixed with 0.5 mL Folin–Ciocalteu reagent and 7 mL water, and then homogenized. The solution was kept at room temperature for 3 min before adding 2 mL of 20% sodium carbonate solution. After an hour in the dark, the absorbance was measured at 732 nm against a blank (solution with no extract added). The calibration curve of gallic acid was used to determine the total phenol content, and the results were reported as mg gallic acid equivalents per gram of dried sample (mg GAE/g).

#### 2.3.3. Total Antioxidant Capacity (TAC) by the Phosphomolybdenum Method

The total antioxidant capacity of the extracts was evaluated by the method of [14]. An aliquot of 0.2 mL of the sample solution was mixed with 4 mL of the reagent solution (0.6 M sulfuric acid, 28 mM sodium phosphate and 4 mM ammonium molybdate). For the blank, 0.2 mL ethanol was mixed with 4 mL of the reagent. The absorbance of the test sample was measured at 695 nm. The antioxidant activity of the samples was expressed as ascorbic acid equivalents for hydrophilic compounds and vitamin E equivalents for lipophilic compounds.

#### 2.3.4. Fatty Acid Determinations

A gas chromatograph Perkin-Elmer Clarus 500 (Massachusetts, United States), fitted with a flame ionization detector (FID) and capillary separation column with a high polar stationary phase TRACE TR-Fame, (Thermo Electron, Massachusetts, United States), with dimensions of 60 m × 0.25 mm × 0.25 μm, was used in order to determine the fatty acid composition of the plants, feeds and yolk samples. Each sample was prepared as described by [15]. The average amount of each fatty acid was used to calculate the sum of the total saturated (SFA), total monounsaturated (MUFA) and total polyunsaturated (PUFA) fatty acids. Indexes of atherogenicity (AI) and thrombogenicity (TI), and the activity of enzymes D5-desaturase and D6-desaturase, were calculated according to [16]:

AI = (C 12:0 + 4 × C 14:0 + C 16:0)/(ΣMUFA + Σ n6 + Σ n3)(1)

TI = (C 14:0 + C 16:0 + C 18: 0)/(0.5 × ΣMUFA + 0.5 × Σ n6 + 3 × Σ n3 + Σ n3/Σ n6)(2)

n6 D5+D6 = [(C 20:2n 6 + C 20:4n6)/(C 18:2n6 + C 20:2n6 + C 20:4n6)] × 100(3)

n3 D5+D6 = [(C 20:5n 3 + C 22:5n3 + C 22:6n3)/(C 18:3n3 + C 20:5n3 + C 22:5n3 + C 22:6n3)] × 100(4)

#### 2.3.5. Extraction Procedure for Analysis of Liposoluble Compounds

The preparation of the plant extracts was carried out using the method previously described by [17]. Before the extraction, saponification was necessary and the samples were hydrolysed with ethanolic potassium hydroxide solution. After putting 2 g of the sample into a 300 mL conical flask, 130 mL ethanol, 100 mg BHT, 2 mL sodium ascorbate solution, 50 mg EDTA and 25 mL 50% potassium hydroxide solution were added. The conical flask was immersed in a water bath with a condenser and boiled for 30 min at 80 °C.

The saponification solution was quantitatively transferred by rinsing with a total volume of 250 mL water into a 1000 mL separating funnel. The saponification flask was rinsed with 25 mL ethanol and 100 mL petroleum ether and transferred to the separating funnel. Extraction with the petroleum ether was repeated four times. The combined extracts were washed with 100 mL water, in order to remove the alkaline traces, until the water remained colorless. The washed extract was then passed through a filter with anhydrous sodium sulphate in order to remove any suspended water, and evaporated under vacuum until dry. The residue was dissolved in 10 mL ethanol.

#### 2.3.6. Lutein and Zeaxanthin Determination

Lutein and zeaxanthin were analyzed using a high performance liquid chromatograph (Perkin Elmer 200 series, Shelton, CT, USA) with a UV detector (445 nm). A stationary phase of 5 µm C18 reversed-phase column (250 × 4.60 mm i.d.) (Nucleodur, Macherey-Nagel, Germany) was used. Chromatographic analysis was carried out under isocratic conditions at a flow rate of 1.0 mL/min and a mobile phase of 13% water and 87% acetone was used.

#### 2.3.7. Vitamin E Determination

Vitamin E determination was performed according to the method described in EC Regulation no. 152/2009, using a high performance liquid chromatograph and a PDA-UV detector (HPLC Finningan Surveyor Plus, Thermo-Electron Corporation, Waltham, MA) at a wavelength of 292 nm. A HyperSil BDS C18 column, with silica gel, dimensions of 250 × 4.6 mm, and a particle size of 5 μm (Thermo-Electron Corporation, Waltham, MA), was used. Chromatographic analysis was carried out under isocratic conditions at a flow rate of 1.5 mL/min and a mobile phase of 4% water, using 96% methanol.

#### 2.3.8. Oxidative Stability Parameters 

Total lipids were extracted using the modified Folch procedure [18]. The minced meat sample (5 g) was homogenized in a 30-mL chloroform/methanol mixture (2:1, v/v). The homogenate was filtered in a separation funnel and 7.5 mL of a 0.88% KCl aqueous solution was added. The sample solution was left to rest for 20 h. The lower organic layer was collected and evaporated at room temperature to a constant weight. The lipid extract resulted from the difference between the initial and final weight of the collector glass.

- Primary oxidation products

The peroxide value (PV) was measured by the ferric thiocyanate method and was expressed as milliequivalents of oxygen per kg lipids (Meq O_2_ kg^−1^) [19]. To the lipid extract sample (0.1 g), 9.9 mL of chloroform/methanol (7:3, v/v) solution was added and the mixture was vortexed. After addition of 50 µL of 10 mmol L^−1^ xylenol orange solution and 50 µL of FeCl_2_ solution (1000 mg kg^−1^), the mixture was left to rest for 5 min at room temperature and then the absorptivity at 560 nm was measured using a V-530 Jasco (Japan Servo Co. Ltd., Japan) spectrophotometer.

The value of conjugated dienes (CD) and trienes (CT) was determined by a spectrophotometric procedure [20] using a lipid extract sample dissolved in 2,2,4-trimethylpentane (iso-octane) and the absorbance values of the sample solution at 233 nm (CD) and 268 nm (CT), which were measured using a V-530 Jasco (Japan Servo Co. Ltd., Japan) spectrophotometer.

- Secondary oxidation products

The *p*-anisidine value was determined by a method based on the reaction between *p*-anisidine and aldehydic compounds present in lipid extract samples in acidic conditions [19]. The lipid extract sample was dissolved in iso-octane and the absorbance of the sample solution was measured at 350 nm. The *p*-anisidine reagent was added to the cuvette, placed in the dark for 10 min and a new spectra was recorded.

The TBARS values were measured according to a method described by [21] using third derivative spectrophotometry with some modifications. TBARS were expressed as milligrams of malondialdehyde (MDA) per kg of muscle (mg MDA kg^−1^). The minced meat sample (5 g) was mixed with 10 mL trichloroacetic acid (7.5%) and 5 mL butyrate hydroxytoluene in ethanol (0.8%). The sample solution was centrifuged at 3000 g for 3 min. Aliquots of 2.5 mL were mixed with 1.5 mL of 0.8% aqueous thiobarbituric acid solution in a test tube and further incubated at 80 °C for 50 min. Following incubation, the sample was cooled under running water and the absorbance was read at 532 nm (sp 0) and 540 nm (sp 3) using a spectrophotometer (Jasco V-530, Japan Servo Co. Ltd., Japan). TBARS values were calculated against a standard curve obtained with 1,1,3,3-tetramethoxypropane (TMP).

### 2.4. Statistics

The experimental data obtained were analyzed using the XLSTAT software (Addinsoft, Paris, France). The following statistical model was used:

Y_ij_ = μ + T_i_ + e_ij_

where Y_ij_ is the mean of the j^th^ observation of the i^th^ treatment; μ is the sample mean; T_i_ is the effect of the i^th^ treatment; and e_ij_ is the effect of error.

The data obtained were analyzed by one way analysis of variance (ANOVA) followed by the Tukey comparison procedure to calculate the interrelation between the groups. A probability level below 5% was considered significant. To verify the relationship between nutrients with antioxidant activity and lipid peroxidation parameters, the Pearson correlation coefficient matrix was used. Differences were significant if *p* < 0.05, and highly significant when *p* < 0.001.

## 3. Results

Table 2 shows the oxidative stability-influencing parameters of dietary supplements and experimental diets. The addition of walnut and bilberry leaves in hens’ diets had a positive effect on TP content, and the vitamin E, lutein and zeaxanthin concentrations of the experimental diets. Total antioxidant capacity was slightly increased in the BL diet under the influence of bilberry leaves (Table 2). The proximate characterization of phytoadditives revealed a 6.76% crude protein content of bilberry leaves and 12.83% for walnut leaves. Regarding crude fat composition, this was 1.38% for bilberry leaves and 2.21% for walnut leaves, and the crude fiber concentrations of the bilberry and walnut leaves were 33.66% and 17.41%, respectively.

### 3.1. The Effects of Bilberry and Walnut Leaves Supplements on the Productive Parameters of Laying Hens

The effect of phytoadditives on the productive parameters of the laying hens during the experimental period is shown in Table 3. The inclusion of dietary bilberry and walnut leaves in laying hens’ diets led to a significant decrease in average egg weight. Supplemental phytoadditives were not significant contributors to average daily feed intake, feed conversion ratio or laying percentage.

### 3.2. The Effects of Bilberry and Walnut Leaves Supplements on the Oxidative Stability-Influencing Parameters of Eggs

Table 4 presents the concentrations of the oxidative stability-influencing parameters in egg yolks collected and analysed at the end of the experiment. The addition of bilberry leaves (BL) and walnut leaves (WL) in laying hens’ diets significantly increased the TP content, and the lutein, zeaxanthin and zinc concentrations in egg yolk samples. The vitamin A concentrations increased under the influence of bilberry leaves compared to the other two groups.

### 3.3. The Effects of Bilberry and Walnut Leaves Supplements on the Fatty Acid Concentrations of Eggs 

Table 5 presents the fatty acid concentrations of eggs collected at the end of the experiment. Significantly increased concentrations of linoleic acid (C 18:2n6), γ linolenic acid (C 18:3n6) and eicosatrienoic acid (C 20:3n6) were noticed for the bilberry leaves-supplemented group and, as a consequence, the sum of n-6 fatty acids was significantly higher compared to the other two groups. For the WL group, only palmitic acid (C 16:0) was significantly decreased compared to the C group.

### 3.4. The Effects of Bilberry and Walnut Leaves Supplements on the Oxidative Stability of Eggs

The oxidative stability parameters of the eggs (Table 6) showed significantly decreased concentrations of primary oxidation products formed in the egg yolks collected from the experimental groups, showing the efficient inhibition effect of phytoadditives on peroxyl radical formation. In addition, a significant effect of supplementing plants on TBARS values was registered compared with the control group, where the degradation process was delayed in eggs from experimental groups.

The correlation matrices (Table 7) between nutrients that impact oxidative processes and lipid peroxidation indices showed a significant (*p* < 0.05 and *p* < 0.01) impact of TP, xantophylls and zinc concentration in egg yolks on the lipid peroxidation parameters.

## 4. Discussion

Walnut leaves are considered to be a rich source of phytochemicals, such as phenolics, flavonoids, and flavonols with free-radical scavenging properties, and are an important exogenous antioxidant source with a role in oxidative stress balance [22]. All groups of phenolic compounds are present in the leaves of bilberry, including high levels of anthocyanins or procyanidins, which have proven anti-radical activity [10]. The walnuts and bilberries are considered significant contributors to carotenoid and tocopherol daily intake [23], but to our knowledge, very little is known about the concentrations of these compounds in leaves. In the present study, significant concentrations of lutein, zeaxanthin and total polyphenols were found in walnut leaves, and the corresponding supplemented diet had over 50% higher concentrations of these compounds compared with the standard diet, and almost 20% more polyphenols.

In this experiment (Table 3), average egg weight was affected by dietary bilberry and walnut leaves supplementation. Anthocyanins are the main polyphenol compounds in berries [24] and they are potentially active against various diseases [25]. Anthocyanins have been reported to alleviate heat stress in chickens, revealing significant effects on growth performance [26]. Studies on effects of blueberry leaf powder on growing pigs productive parameters, showed that body weight or average daily gain were not significantly affected [27]. Studies on chickens have demonstrated the effectiveness of walnut leaves in enhancing growth [28], in contrast with our results where walnut leaves dietary supplements did not improve the productive parameters.

In the present study, dietary bilberry and walnut leaves supplements improved the antioxidant bioactive compounds (TP, vitamin A, lutein, zeaxanthin and zinc) of egg yolks (Table 4). Phenolics are a major phytochemical class, which includes chemical compounds with one or more phenolic groups [29]. Under natural conditions, deposition of simple phenolic acids into the chicken egg yolk is very limited due to its hydrophilic nature [30]. In a study of the effect of dried tomato peel on laying hens’ yolk carotenoids and phenols [31], the authors showed that different amounts of phytoadditives increased the total phenol content, which is well correlated with cholesterol reduction in yolk.

Lutein and zeaxanthin cannot be synthetised by hens, so the carotenoids stored in the yolk are related to the diet [32]. Lutein and zeaxanthin exhibit antioxidant properties and were found to be important compounds in the prevention of age-related macular degeneration [32]. Vegetables and fruits are important dietary sources of carotenoids but their bioavailability it is considered to be poor, in contrast with xanthophyll from egg yolks, which has been demonstrated to have a higher bioavailability [2]. More studies were conducted with the aim to improve the carotenoid profile of egg yolks. Natural dietary xanthophyll sources used in experiments on laying hens are corn and corn gluten meal, lucerne, marigold, red pepper [33] and *Chlorella* [34]. In our study, at the end of the experiment, significantly increased concentrations of xanthophyll in the egg yolks of experimental groups were found compared to the control group, reflecting the concentration of carotenoids in the diet. Lutein is a major carotenoid in the hens’ diet and the egg yolks, and it is transferred from the feed to the egg yolk with high efficiency depending on the carotenoid composition and content of the diet [35].

Our results showed significantly increased vitamin A and only increased trend of vitamin E concentrations in the BL group compared to the C group. In contrast with our findings, some researchers considered that the competition for the binding sites that occurs between vitamins leads to an adverse effect of vitamin A on the absorption and metabolism of vitamin E and carotenoids [36]. On the other hand, egg yolk is a lipophilic matrix and tocopherol has a protective effect on carotenoids and reduces their oxidative degradation during storage [37].

Zinc does not directly interact with reactive oxygen species. It plays an important role in antioxidant defense by activation of antioxidant enzymes, inhibiting some important pro-oxidant enzymes, competing with redox-active transition metals such as iron and copper for certain binding sites, protecting the sulfhydryl groups of proteins from oxidation, or forming metallothionein, which is a scavenger of radicals [38]. The increasing zinc concentrations in yolks with bilberry and walnut leaves supplements is an important parameter of antioxidant system for eggs.

The concentration of linoleic acid (C 18:2n6) was significantly increased in the BL group compared with the other two groups (Table 5), but the concentration of arachidonic acid (C 20:4n6), the main product of desaturase/elongase enzyme activity, was not different between groups. The competition between linoleic acid and α linolenic acid is responsible for synthesis of PUFA. Linoleic acid is easily incorporated into tissues but α linolenic acid inhibits the conversion reaction from linoleic acid to arachidonic acid by elongation and desaturation [39]. No significant differences in the desaturase enzyme activity, estimated as the ratio between products (long chain fatty acids from n-3 and n-6 series) and precursors (α linolenic acid and linoleic acid) [16], were noticed between groups.

Atherogenicity index (AI) and thrombogenicity index (TI) are the main parameters of animal fat used to assess the nutritional value and the impact on consumer health [4,40]. They represent the relationship between saturated fatty acids, considered pro-atherogenic and pro-thrombogenic lipids and unsaturated fatty acids, considered anti-atherogenic and anti-thrombogenic lipids [4,41]. In our study, AI were not influenced by bilberry or walnut leaves supplements present in laying hens’ diets, while TI was significantly decreased in the WL group compared with the control, showing a beneficial effect on human health.

Inclusion of bilberry and walnut leaves in the diet promotes the enrichment of antioxidant compounds in egg yolks. The antioxidant compounds of eggs (polyphenols, vitamins, minerals) probably act synergistically with each other, providing a protective effect against lipid peroxidation and increasing the oxidative stability of yolk lipids. Under the influence of bilberry leaves supplements, the concentrations of CD and CT (primary oxidation products) and TBARS values (secondary oxidation products) decreased compared with the control group. The indicators of the initial stage of oxidation (PV and CD) decreased for the WL group compared with the control group, as did the amount of alkenals, alcadienals and aldehides generated during decomposition of hydroperoxides, measured as *p*-anisidine and TBARS values.

Lipid peroxidation of egg yolks is a relevant free radical chain reaction, which increases during processing, storage and exposure to light [42]. Polyunsaturated fatty acids are readily attacked by free radicals, being oxidized into lipid peroxides [43]. An inverse relationship between antioxidant concentrations in the diet and egg yolk lipid peroxidation parameters has been reported in previous research studies [3,44], but the antioxidant effect of bilberry or walnut leaves on the quality of eggs is lacking. A study on diabetic Wistar rats revealed that mulberry anthocyanins reduce liver TBARS levels [45]. A study on the oxidative stability of meat showed that walnut leaf powder can be used as a natural antioxidant in ground pork products [46].

This study of nutrients with antioxidant activity and lipid peroxidation parameters revealed a significant correlation between the primary oxidation products (PV, CD and CT) and TP content of egg yolks (Table 7), which means that polyphenols are the major contributor to the delay in hydroperoxide formation in the initial phase of peroxidation. Lutein, zeaxanthin and zinc were significantly correlated with *p*-anisidine and TBARS, being the bioactive compounds that inhibit the formation of secondary oxidation products.

## 5. Conclusions

The results of the present study showed that the active ingredients contained in bilberry and walnut leaves included in laying hens’ diets (0.5 and 1% respectively, inclusion rate) enhanced the oxidative stability of egg yolks and retarded the lipid peroxidation process during storage.

## Figures and Tables

**Table 1:** Composition and chemical analyses of the basal diets.

Ingredients.	C Group (%)	BL (%)	WL (%)
Corn	30	29.5	29
Wheat	31.46	31.46	31.46
Gluten	4	4	4
Soybean meal	21.2	21.2	21.2
Vegetable Oil	1.46	1.46	1.46
Lysine	0.06	0.06	0.06
Methionine	0.13	0.13	0.13
Calcium carbonate	8.78	8.78	8.78
Monocalcium phosphate	1.46	1.46	1.46
Salt	0.4	0.4	0.4
Choline	0.05	0.05	0.05
Billberry leaves	0	0.5	0
Walnut leaves	0	0	1
Premix ^1^	1	1	1
Total	100	100	100
*Calculated analysis, %*			
Dry matter	88.06	89.12	88.64
M.E. poultry, (kcal/kg)	2800	2800	2800
Crude protein	17.80	17.78	17.82
Crude fat	2.94	2.93	2.94
Crude fiber	4.06	4.21	4.20
Calcium	3.90	3.90	3.90
Available phosphorous	0.42	0.42	0.42
Lysine	0.87	0.87	0.87
Methionine	0.44	0.44	0.44
Met + cis	0.78	0.78	0.78
Threonine	0.58	0.58	0.58
Triphtopan	0.18	0.18	0.18

^1^ Content per kg diet: vitamin A: 13,500 IU; vitamin D3: 3000 IU; vitamin E: 27 mg; vitamin K3: 2 mg; vitamin B1: 2 mg; vitamin B2: 4.8 mg; pantothenic acid: 14.85 mg; nicotinic acid: 27 mg; vitamin B6: 3 mg; vitamin B7: 0.04 mg; vitamin B9: 1 mg; vitamin B12: 0.018 mg; vitamin C: 25 mg; manganese: 71.9 mg; iron: 60 mg; copper: 6 mg; zinc: 60 mg; cobalt: 0.5 mg; iodine: 1.14 mg; selenium: 0.18 mg.

**Table 2:** The oxidative stability-influencing parameters of dietary supplements and experimental diets.

	Bilberry Leaves	Walnut Leaves	C	BL	WL
TAC (mM equiv asc acid) *	301.46	254.29	36.37	40.72	35.86
TAC (mM equiv vit E) *	184.15	141.15	30.87	34.98	30.52
TP (mg/g) *	51.73	56.75	2.13	2.51	2.55
Vitamin E (mg/kg)	111.28	157.54	38.73	48.15	41.16
Lutein and Zeaxanthin (mg/kg)	70.59	264.09	8.18	9.42	12.99
Zinc (mg/kg)	43.88	32.64	103.66	103.96	104.53

* TAC - total antioxidant capacity; TP - total polyphenols.

**Table 3:** The effect of supplemental bilberry and walnut leaves on the productive parameters of the laying hens.

	C	BL	WL	SEM	*p* Value
Initial Body Weight (kg)	1.71	1.72	1.72	0.054	0.6256
Final Body Weight (kg)	1.78	1.78	1.79	0.068	0.8259
Average Daily Feed Intake (g/hen)	108.29	103.18	105.81	0.777	0.7865
Feed Conversion Ratio (kg feed/kg egg)	1.83	1.86	1.85	0.021	0.5557
Egg Weight (g)	60.43 ^a^	58.02 ^c^	58.99 ^b^	0.153	<0.0001
Laying Percentage	98.22	96.07	98.21	0.752	0.0731

Means within a row with no common superscript differ (*p* < 0.05).

**Table 4:** The oxidative stability-influencing parameters in egg yolks, collected at the end of the experiment.

	C	BL	WL	SEM	*p* Value
TAC (mM equiv AA) *	5.10	6.44	6.05	0.412	0.1011
TAC (mM equiv vit E) *	4.38	5.65	4.67	0.399	0.1014
TP (mg/g) *	0.94^b^	1.03^a^	1.02^a^	0.015	0.0022
Vitamin E (mg/kg)	105.12	118.46	106.94	4.770	0.1430
Vitamin A (mg/kg)	10.02^b^	12.61^a^	11.09^b^	0.625	0.0218
Lutein and Zeaxanthin (mg/kg)	9.60^b^	12.06^a^	13.14^a^	0.392	0.0001
Zinc (mg/kg)	79.10^b^	81.96^a^	81.92^a^	0.419	0.0005

^a,b^ Means within a row with no common superscript differ (*p* < 0.05). * TAC: total antioxidant capacity; TP: total polyphenols.

**Table 5:** The fatty acid composition of egg yolks, collected at the end of the experiment.

	C	BL	WL	SEM	*p* Value
C 14:0	0.36	0.,34	0.33	0.0241	0.6349
C 14:1	0.08	0.08	0.08	0.0046	0.9580
C 15:0	0.07 ^a^	0.07 ^ab^	0.06 ^abc^	0.0052	0.4813
C 15:1	0.09	0.10	0.11	0.0149	0.5782
C 16:0	25.98 ^a^	25.36 ^ab^	24.82 ^b^	0.1892	0.0023
C 16:1	3.71 ^a^	3.35 ^b^	3.47 ^ab^	0.0949	0.0456
C 17:0	0.11	0.14	0.13	0.0140	0.2480
C 17:1	0.10	0.08	0.08	0.0110	0.2309
C 18:0	10.66 ^b^	10.78 ^b^	10.89 ^b^	0.2637	0.8314
C 18:1	33.76	33.55	34.88	0.3845	0.0583
C 18:2n6	16.39 ^b^	17.53 ^a^	16.44 ^b^	0.0782	<0.0001
C 18:3n6	0.13 ^b^	0.14 ^a^	0.14 ^ab^	0.0048	0.0505
C 18:3n3	0.59	0.66	0.57	0.0294	0.1077
C 20:2n6	0.16	0.19	0.18	0.0159	0.2728
C 20:3n6	0.26 ^b^	0.32 ^a^	0.27 ^b^	0.0108	0.0039
C 22:1n9	0.11	0.12	0.12	0.0083	0.7582
C20:3n3	0.31	0.33	0.27	0.0254	0.2379
C 20:4n6	3.88	3.89	3.90	0.1382	0.9939
C 24:1n9	0.24	0.25	0.28	0.0103	0.1093
C 22:4n6	0.96	0.85	0.98	0.0394	0.0803
C 22:5n3	0.14	0.15	0.14	0.0079	0.4040
C 22:6n3	1.68	1.68	1.79	0.0641	0.3921
Other Fatty Acids	0.23 ^a^	0.02 ^b^	0.08 ^b^	0.0264	0.0002
TOTAL					
Σ SFA *	37.17	36.69	36.22	0.3317	0.1638
Σ MUFA *	38.10	37.53	39.02	0.4577	0.1016
Σ n-3	2.71	2.82	2.77	0.0651	0.4906
Σ n-6	21.78 ^b^	22.93 ^a^	21.91 ^b^	0.1736	0.0005
Ratio n-6/n-3	8.05	8.12	7.92	0.1523	0.6477
					
AI *	0.61	0.60	0.58	0.0083	0.0992
TI *	0.97 ^a^	0.94 ^ab^	0.92 ^b^	0.0107	0.0395
n3 D5 + D6	75.49	73.48	77.05	1.3323	0.1992
n6 D5 + D6	19.73	18.89	19.88	0.5576	0.4202

^a,b,c^ Means within a row with no common superscript differ (*p* < 0.05). * SFA: saturated fatty acids; MUFA: monounsaturated fatty acids; AI: atherogenicity index; TI: thrombogenicity index.

**Table 6:** Effect of dietary phytoadditives on egg yolk oxidative stability during storage.

	C	BL	WL	SEM	p Value
Primary Oxidation Products					
PV * (meq active O_2_/kg)	0.17 ^a^	0.14 ^ab^	0.13 ^b^	0.0097	0.0250
CD * (µmol/g)	7.54 ^a^	5.02 ^b^	3.75 ^b^	0.3623	0.0001
CT * (µmol/g)	2.29 ^a^	1.46 ^b^	1.34 ^ab^	0.1530	0.0383
Secondary Oxidation Products					
p-Anisidine	6.42	4.57	4.05	0.6984	0.0914
TBARS (mg/kg)	0.15 ^a^	0.13 ^b^	0.12 ^b^	0.0052	0.0187

^a,b^ Means within a row with no common superscript differ (*p* < 0.05) * PV: peroxide value; CD: conjugated dienes; CT: conjugated trienes; *p*-anisidine: para anisidine; TBARS: thiobarbituric acid reactive substances.

**Table 7:** Correlations between nutrients with antioxidant activity and lipid peroxidation parameters.

	TP	Vit E	Vit A	Lutein	Zn	PV	CD	CT	P Anis	TBARS
TP ***	1.00	0.41	0.17	0.65 **	0.77 *	−0.63 **	−0.66 **	−0.45	−0.39	−0.48
Vit E		1.00	0.49	0.05	0.10	−0.12	−0.19	−0.20	−0.32	−0.14
Vit A			1.00	0.01	0.09	−0.19	−0.11	−0.20	−0.10	−0.19
Lutein				1.00	0.78 *	−0.65 **	−0.72 **	−0.42	−0.63 **	−0.64 **
Zn					1.00	−0.61 **	−0.52 **	−0.46	−0.55 **	−0.40
PV ***						1.00	0.70 **	0.50	0.62 **	0.54 **
CD ***							1.00	0.70 *	0.45	0.66 **
CT ***								1.00	0.35	0.62 **
P Anis ***									1.00	0.59 **
TBARS										1

* Values in bold are different from 0 with a significance level alpha = 0.001; ** Values in bold are different from 0 with a significance level alpha = 0.05; *** TP: total polyphenols; PV: peroxide value; CD: conjugated dienes; CT: conjugated trienes; p anis: para anisidine; TBARS: thiobarbituric acid reactive substances.
